# Retraction Note: The association between neutrophil—lymphocyte ratio and poor outcomes following infant cardiac surgery

**DOI:** 10.1186/s12872-024-04285-0

**Published:** 2024-10-26

**Authors:** Peng Gao, Jinping Liu, Xu Wang, Peiyao Zhang, Yu Jin, Liting Bai, Yixuan Li

**Affiliations:** Pediatric Cardiac Surgery Center, Fuwai Hospital, National Center for Cardiovascular Diseases, Chinese Academy of MedicalSciences and Peking Union Medical College, No. 167, North Lishi Road, Xicheng District, 100037 China

**Retraction Note: BMC Cardiovascular Disorders (2021) 21:529**

10.1186/s12872-021-02345-3

The Editor has retracted this article because, owing to an administration error in the editorial office, it was published before the peer review process had been undertaken. Post-publication peer review raised concerns that cannot be easily addressed by post-publication corrections as additional work is required. The authors have been invited to submit a new revised manuscript that will undergo robust peer review. The Publisher apologises to the authors for the administrative error. None of the authors has responded to correspondence from the Editor about this retraction.

